# Preparation of Corn Peptides with Anti-Adhesive Activity and Its Functionality to Alleviate Gastric Injury Induced by *Helicobacter pylori* Infection In Vivo

**DOI:** 10.3390/nu15153467

**Published:** 2023-08-05

**Authors:** Guanlong Li, Xiaolan Liu, Zhengfei Miao, Nan Hu, Xiqun Zheng

**Affiliations:** 1 Heilongjiang Provincial Key Laboratory of Corn Deep Processing Theory and Technology, College of Food and Bioengineering, Qiqihar University, Qiqihar 161006, China; 03580@qqhru.edu.cn (G.L.); 2021907279@qqhru.edu.cn (Z.M.); 03564@qqhru.edu.cn (N.H.); 2 College of Food Science, Heilongjiang Bayi Agricultural University, Daqing 163319, China; zhengxiqun@byau.edu.cn

**Keywords:** corn protein-derived peptides, *Helicobacter pylori*, anti-adhesive activity, anti-inflammatory, gastric injury

## Abstract

More than 50% of the world population is infected with *Helicobacter pylori* (*H. pylori*), which is classified as group I carcinogen by the WHO. *H. pylori* surface adhesins specifically recognize gastric mucosal epithelial cells’ (GES-1 cells) receptor to complete the adhesion. Blocking the adhesion with an anti-adhesion compound is an effective way to prevent *H. pylori* infection. The present study found that corn protein hydrolysate, hydrolyzed by Neutral, effectively alleviated gastric injury induced by *H. pylori* infection through anti-adhesive and anti-inflammatory effects in vitro and in vivo. The hydrolysate inhibited *H. pylori* adhesion to GES-1 cells significantly, and its anti-adhesive activity was 50.44 ± 0.27% at 4 mg/mL, which indicated that the hydrolysate possessed a similar structure to the GES-1 cells’ receptor, and exhibited anti-adhesive activity in binding to *H. pylori*. In vivo, compared with the *H. pylori* infection model group, the medium and high dose of the hydrolysate (400–600 mg/kg·bw) significantly decreased (*p <* 0.05) the amount of *H. pylori* colonization, pro-inflammatory cytokines (IL-6, IL-1β, TNF-α and MPO), chemokines (KC and MCP-1) as well as key metabolites of NF-κB signaling pathway levels (TLR4, MyD88 and NF-κB), and it increased antioxidant enzyme contents (SOD and GSH-Px) and the mitigation of *H. pylori*-induced pathological changes in the gastric mucosa. Taken together, these results indicated that the hydrolysate intervention can prevent *H. pylori*-induced gastric injury by anti-adhesive activity and inhibiting the NF-κB signaling pathway’s induction of inflammation. Hence, the corn protein hydrolysate might act as a potential anti-adhesive agent to prevent *H. pylori* infection.

## 1. Introduction

*Helicobacter pylori* (*H. pylori*) is able to colonize the human gastric mucosa, which is a Gram-negative, spiral-shaped, and microaerophilic bacterium. It is reported that more than 50% of the world population has been infected with *H. pylori*, and the current infection rate in China is 60–70%, which has been classified as a group I carcinogen by the World Health Organization (WHO) [1]. *H. pylori* specifically adheres to gastric mucosal epithelial cells (GES-1 cells); it is recognized as one of the important causative agents of peptic ulcers, chronic gastritis and other gastric disorders; and it is also closely associated with the development of gastric cancer. *H. pylori* infection causes oxidative stress and neutrophil infiltration in gastric tissue, which promotes levels of pro-inflammatory cytokines and chemokines. In addition, overexpression of pro-inflammatory cytokines activates the NF-κB signaling pathway, which is one of the major pathways inducing gastric cell inflammation [2]. To date, antibiotics are the most widely used agents for the treatment of *H. pylori* infection, with the eradication rates ranging from 60–90% [3]. Although antibiotics have good effects, they still possess some side effects and resistance to *H. pylori*; extensive research has been conducted to develop alternative approaches to prevent and treat *H. pylori* [4], and anti-adhesion of *H. pylori* is an effective strategy.

Adhesion proteins, outer membrane proteins (OMPs) of the *H. pylori*, could specifically recognize receptors on the GES-1 cells’ surface to achieve colonization of *H. pylori* [5]. Many adhesins are expressed by *H. pylori*, of which sialic acid-binding adhesin (SabA) and Lewis-b blood group antigen-binding adhesin (BabA) are dominant adhesins. SabA and BabA specifically recognize sialyl-Lewis-a and Lewis-b, respectively [6]. Anti-adhesion therapy, a promising strategy, could effectively prevent infection by inhibiting *H. pylori* adhesion to GES-1 cells [7]. Specifically, the anti-adhesion component acts as GES-1 cell receptor analogs or *H. pylori* surface adhesin analogs to block the adhesion between the *H. pylori* and the GES-1 cells [8]. Recent studies found that a variety of natural products possessed anti-adhesive effects to prevent *H. pylori* infection in vitro, such as pea peptides [9], pectin from apples [10], polyphenols from cranberries [11], 3′-sialyllactose [12] and wheat germ-derived peptides [8]. Despite the above natural products showing anti-adhesive activities in vitro, their anti-adhesion effects were not evaluated in vivo.

Corn gluten meal (CGM) is a co-product of the wet milling process; its total protein content is 60–70% (*w*/*w*), including zein, glutenin, globulin and albumin [13]. Due to CGM’s unique amino acid composition and partial amino acid sequence, it is also a quality protein source for preparing bioactive peptides [14]. By enzymatic hydrolysis, the solubility of corn protein is elevated significantly, and the functional sequences may be released. The reported major bioactive peptides from CGM included antioxidant activity [15], antagonism alcohol-induced liver injury [16] and angiotensin converting enzyme inhibitory activity [17]. In the literature available, no reports could be found about corn protein-derived peptides with anti-adhesive activity preventing *H. pylori* infection. The aim of this study is to effectively prepare corn protein-derived peptides with anti-adhesive activity, evaluate their functionality to alleviate gastric injury induced by *H. pylori* infection in vivo and investigate the possible underlying mechanisms.

## 2. Materials and Methods

### 2.1. Materials and Chemicals

Corn gluten meal (CGM) was purchased from FuFeng Development Co., Ltd. with a total protein content of 60.44% (*w*/*w*) (Qiqihar, Heilongjiang, China). Neutral (21,000 U/mL), Alcalase (23,000 U/g), Protamex (38,000 U/g), Trypsin (21,000 U/g) and Papain (11,000 U/g) were obtained from Novo Nordisk (Bagsvaerd, Denmark). Protease P (48,000 U/g), Protease N (48,000 U/g) and Protease M (61,000 U/g) were purchased from Amano Pharmaceutical Co., Ltd. (Nagoya, Japan). Dimethyl sulfoxide (DMSO) and Fluorescein isothiocyanate (FITC) were bought from Solarbio Life Sciences (Beijing, China). *H. pylori* (43,504) was bought from ATCC. GES-1 cells were purchased from JianCheng Co., Ltd. (Nanjing, Jiangsu, China). Fetal bovine serum (FBS) and Dulbecco’s modified eagle medium (DMEM) were bought from GE Healthcare (Pittsburgh, PA, USA).

### 2.2. Starch Removal of CGM 

The starch was removed from the CGM as previously described [14]. Briefly, CGM was dispersed in distilled water (10%, *w*/*v*), adjusted pH to 6.5 with 0.1 M HCl, then α-amylase (30 U/g of protein) was added and incubated at 65 °C for 120 min. Next, the mixture was heated to 95 °C for 5 min to inactivate the enzyme and centrifuged at 4000× *g* for 15 min. The precipitate was washed three times with distilled water, and the pretreated CGM was obtained by drying for preparing the corn protein hydrolysates.

### 2.3. Preparation of Corn Protein Hydrolysates

The pH of the 15% suspension (*w*/*v*, protein/water) of pretreated CGM was adjusted to the optimum pH of protease with 0.1M HCl. Enzymatic hydrolysis of the pretreated CGM was catalyzed at the optimum temperature and pH of all the proteases (Table 1). The hydrolysis time was 150 min, and the enzyme/substrate ratio (U/g·protein) was 400 U/g for all the proteases. Following the hydrolysate reaction, the hydrolysate mixture was heated to 95 °C for 10 min and then centrifuged at 4000× *g* for 15 min. The hydrolysate was collected and lyophilized to be used for the determination of anti-adhesive activity.

### 2.4. H. pylori and GES-1 Cell Line Culture Conditions

The culture conditions of *H. pylori* ATCC 43,504 and GES-1 cells were as described in previous report [8].

### 2.5. Labeling of H. pylori with FITC

An amount of 2 mg FITC was dissolved in 1 mL of DMSO, then the FITC solution was sterilized with a 0.22 μm filter. The *H. pylori* suspension and the FITC solution were mixed (1:1, *v*/*v*) and incubated in the dark for 30 min for the combination to occur. The *H. pylori* was precipitated to end the labeling operation (4500× *g*, 3 min) and washed three times with 0.1 M PBS buffer (pH 7.4) to remove free FITC. Finally, the FITC-labeled *H. pylori* solution was diluted with Medium no. 18 until the OD_600_ value was about 0.1 (10^8^ cfu/mL), which was further used in the anti-adhesion assay.

### 2.6. Anti-Adhesion Assay

**Figure 1:**
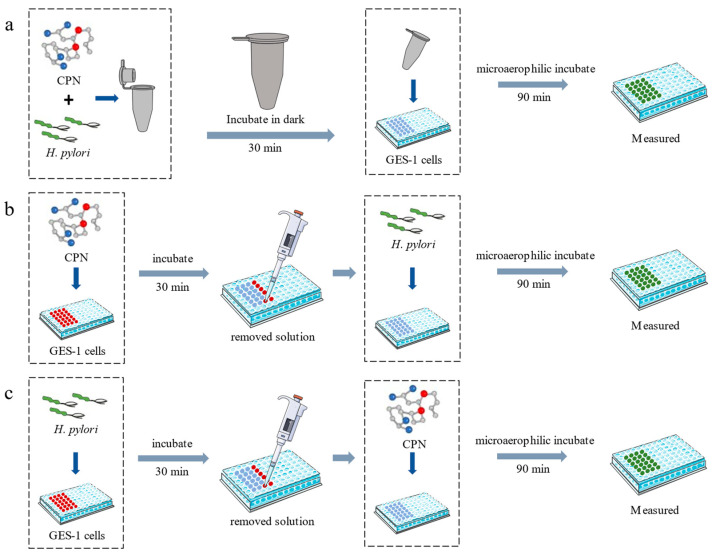
The anti-adhesion assays of CPN. (**a**) CPN and *H. pylori* were pre-incubated; (**b**) CPN and GES-1 cells were pre-incubated; (**c**) *H. pylori* and GES-1 cells were pre-incubated.

The anti-adhesive activity was measured as described in the previous report with minor modifications [8]. The anti-adhesion assay was performed in three different manners (Figure 1) and involved Neutral corn protein hydrolysate (CPN), *H. pylori* and GES-1 cells. CPN was treated with a 0.22 μm filter for sterilization. (1) CPN (8 mg/mL) and FITC-labeled *H. pylori* (10^8^ cfu/mL) were mixed together (1:1, *v*/*v*) and incubated for 30 min in the dark. Then, 100 μL of the *H. pylori*-CPN solution was added to the GES-1 cells inside the 96-well plate (3 × 10^5^ cells/well), then the 96-well plate was incubated at 37 °C for 1.5 h under microaerophilic conditions (5% O_2_, 10% CO_2_ and 85% N_2_). (2) An amount of 100 μL of CPN was added to each well with GES-1 cells and incubated at 37 °C for 0.5 h under microaerophilic conditions, then the CPN solution was removed and 100 μL of FITC-labeled *H. pylori* was added to the GES-1 cells at the same conditions and incubated for 1.5 h. (3) An amount of 100 μL of FITC-labeled *H. pylori* was added to the GES-1 cells and incubated at 37 °C for 0.5 h under microaerophilic conditions. After the FITC-labeled *H. pylori* solution was removed, 100 μL of CPN solution was added to the GES-1 cells, then it was incubated under microaerophilic conditions for 1.5 h.

The treated GES-1 cells in the above three manners were washed with 200 μL of 0.1 M sterile PBS buffer (pH 7.4) three times to remove free *H. pylori*, then 100 μL of 0.1 M PBS buffer (pH 7.4) was added to the 96-well plate. Finally, the fluorescence intensity of the 96-well plate was measured at the excitation wavelength of 485 nm and emission wavelength of 530 nm. The standard curve was set up by the value of OD_600_ and fluorescence intensity, and then the concentration of *H. pylori* was calculated. The CPN was replaced by 100 μg/mL of rebamipide and DMEM incomplete medium as the positive control and negative control, respectively.

(1) $$Inhibition\left(\%\right)=\frac{number\;of\;H.\;pylori\;negative\;control-number\;of\;H.\;pylori\;sample}{number\;of\;H.pylori\;negative\;control}\times 100$$

The percent inhibition (anti-adhesive activity) of CPN was calculated as follows:

### 2.7. Animals and Experimental Design

Six-week-old male KM mice were obtained from the Changchun Yisi Experimental Animal Research Center (Changchun, China). The animal certificate number is SCXK (Ji)-2018–0007. The animal study protocol was approved on 8 June 2021 by the Animal Ethics Committee of the College of Food and Bioengineering, Qiqihar University (Approval No. 2021-004). The temperature and humidity conditions were 22 ± 1 °C and 55 ± 5%, respectively, and the mice were fed with free access to water and food. 

After acclimation for 1 week, mice were randomly divided into six groups (*n* = 8). Normal control (NC), *H. pylori* infection model (HM), 200 mg/kg·bw CPN group (CPN-200), 400 mg/kg·bw CPN group (CPN-400), 600 mg/kg·bw CPN group (CPN-600) and positive group (14.25 mg/kg amoxicillin +7.15 mg/kg Clarithromycin + 14.2 mg/kg Metronidazole). Body weight was measured once per day during the experimental period. 

At 1–2 weeks, NC and HM groups were given 0.3 mL 0.9% normal saline and the positive group was given 0.3 mL the mixed antibiotics, whereas the CPN groups were given 200 mg/kg·bw, 400 mg/kg·bw and 600 mg/kg·bw CPN, respectively. 

At 3–4 weeks, all groups continued to be given different doses of CPN or 0.9% normal saline or antibiotics. Except NC group, other groups were given 0.4 mL *H. pylori* (about 10^9^ cfu/mL) after 30 min. The *H. pylori* was given every other day, 7 times in total. 

At 5 weeks, all groups continued to be given different doses of CPN or 0.9% normal saline or antibiotics.

### 2.8. Analysis of H. pylori Enumeration of Gastric Tissue

The sterile gastric homogenate serially diluted in 0.1 M PBS buffer, 100 μL of each dilution, was spread-plated onto Medium no. 260, then the plates were incubated at 37 °C for 48 h under microaerophilic conditions. The colonies were counted and expressed as CFU/g gastric tissue.

### 2.9. Measurement of Biochemical Parameters by ELISA

The mice were fed in the experiment for five weeks. The mice were fasted for 12 h before being sacrificed through cervical dislocation. After the gastric tissue was weighed, it was split into two sections: one for histopathologic study, the other for homogenization in ice-cold 0.9% normal saline to generate a 10% gastric homogenate under aseptic conditions.

According to the explanatory memorandum, tumor necrosis factor-α (TNF-α), malondialdehyde (MDA), monocyte chemoattractant protein-1 (MCP-1), Superoxide dismutase (SOD) activities, lactate dehydrogenase (LDH), toll-like receptor 4 (TLR4), GSH peroxidase (GSH-Px) activities, interleukin (IL)-1β, myeloperoxidase (MPO) activity, keratinocyte chemokine (KC), interleukin (IL)-6, myeloid differentiation factor88 (MyD88) and NF-κB levels in the gastric homogenates were measured by enzyme-linked immunosorbent assay kits (JiangLai bio, Shanghai, China).

### 2.10. Histopathologic Analysis

The gastric tissue samples were fixed in 10% buffered formalin solution, and then were embedded in paraffin. The 4 μm tissue sections were made, then the sections were stained with hematoxylin and eosin and visualized using microscope (Olympus BX43 microscope).

### 2.11. Statistical Analysis

All results were expressed as mean ± standard deviations (SD), and ANOVA and Tukey’s tests were used to analyze the statistical comparisons. SPSS statistics 19 (SPSS Inc., Chicago, IL, USA) was used to analyze the data. Significant differences were considered at *p* < 0.05.

## 3. Results and Discussion

### 3.1. Corn Protein-Derived Peptides Preparation

CGM contains mainly zein (68%) and glutelin (22%) [13]. Zein has a high ratio of hydrophobic amino acids (Leu (20%), Ala (10%), Pro (10%)) [14], leading to a high frequency of hydrophobic amino acid residue sequences in its protein primary structure. For example, α-zein (22 kDa) has some typical fragments, such as LLAL_11–14_, APIA_30–33_, LLPP_35–38_, AIAA_61–64_ and LAAA_181–184_ (Table 2). Corn glutelin has a high glutamine (Gln, Q) content of 33% [14] and many sequences of QQ or QQQ located in its primary structure, for example, QQ_104–105,153–154,157–158,199–200,208–209_ and QQQ_181–183_ in glutelin (26.3 kDa) (Table 2).

Alcalase, Protamex and Protease M are endoproteases; they act on hydrophobic and aromatic amino acid residues, such as Ala, Leu and Phe. Protease N and Protease P mainly act on hydrophobic and hydroxyl amino acids, such as Thr, Ser and Pro. Neutral is also an endoprotease, and it mainly hydrolyzes the aromatic amino acids. According to the structural characteristics of corn protein, and taking into account the substrate specificity of the proteases, the above proteases were chosen to hydrolyze corn protein, expecting to obtain corn protein hydrolysates with anti-adhesive activity. Thus, the effect of the protease on the anti-adhesive activity of the hydrolysates was investigated. Eight proteases were applied for producing anti-adhesive hydrolysates from CGM at the enzyme/substrate ratio of 400 U/g·protein and hydrolysis time of 120 min.

As shown in Table 1, the anti-adhesive activities of different protease hydrolysates had significant differences (*p* < 0.05). All hydrolysates, except the hydrolysate hydrolyzed by papain, showed anti-adhesive activities, and the hydrolysate (CPN) hydrolyzed by Neutral had the best anti-adhesive activity (50.44 ± 0.27%) at the concentration of 4 mg/mL; hydrolysates hydrolyzed by Protease N and Protease P also showed better anti-adhesive activities. It was speculated that hydrophobic and aromatic amino acids in the peptides had an important effect on the anti-adhesive activity of the hydrolysates.

Moreover, trypsin and papain hydrolysates showed the lowest anti-adhesive activities. Trypsin and papain have similarity in the cleavage of proteins, and they could act on basic amino acid residues such as Arg and Lys; it is possible that Arg and Lys have less of an effect on the anti-adhesive activity of hydrolysates. This finding was consistent with Sun et al. [8], where it was also found that the trypsin hydrolysate had low anti-adhesive activity.

### 3.2. Assay of Anti-Adhesive Activity of CPN

*H. pylori* could specifically adhere to the GES-1 cells. Adhesion proteins, OMPs of *H. pylori*, specifically recognize receptors of GES-1 cells’ surface to achieve *H. pylori* infection [5]. Anti-adhesion therapy, a promising strategy, could effectively prevent infection by inhibiting *H. pylori* adhesion to GES-1 cells. Specifically, the anti-adhesion component acts as GES-1 cell receptor analogs or *H. pylori* surface adhesin analogs to block the adhesion between the *H. pylori* and the GES-1 cells. 

To analyze the possible anti-adhesive mechanism of CPN, three inhibition assays were performed in the present study. CPN (as receptor analogs) and *H. pylori*, CPN (as adhesin analogs) and GES-1 cells, and *H. pylori* and GES-1 cells were mixed, respectively. When CPN and *H. pylori* were pre-mixed (Figure 1a) and incubated for 0.5 h in dark, then the CPN-*H. pylori* solution was added to the 96-well plate with GES-1 cells (3 × 10^5^ cells/well) and incubated at 37 °C for 1.5 h under microaerophilic conditions, it was found excitedly that the measured fluorescence intensity of the CPN group decreased sharply, compared with that of control group, indicating that the number of FITC-labeled *H. pylori* adhering to the GES-1 cells’ surface significantly reduced. The anti-adhesive activity of CPN was 50.44 ± 0.27%, showing that CPN was able to block the *H. pylori* adhesion to GES-1 cells as receptor analogs; this might be a possible mechanism. 

When CPN and GES-1 cells were pre-mixed (Figure 1b) and incubated for 0.5 h under microaerophilic conditions, then the solution was removed and the FITC-labeled *H. pylori* was added to each well and incubated for 1.5 h, the fluorescence intensity of each well was measured. Compared with that of control group, it was found that the fluorescence intensity of the CPN group did not change significantly, showing that the number of FITC-labeled *H. pylori* adhering to the GES-1 cells’ surface did not reduce. CPN did not exhibit anti-adhesive activity, suggesting that CPN could not inhibit the adhesion of *H. pylori* as an adhesion analog. 

When *H. pylori* and GES-1 cells were pre-mixed (Figure 1c) and incubated at 37 °C for 0.5 h under microaerophilic conditions, then after the solution was removed, CPN was added to GES-1 cells and incubated for 1.5 h, it was found that the fluorescence intensity of the CPN group did not change significantly, compared with that of control group, and CPN did not remove *H. pylori* that was already bound to GES-1 cells, indicating that CPN could not destroy the binding between GES-1 cells and *H. pylori*. 

Based on the above, the CPN could inhibit the adhesion of *H. pylori*, when used as receptor analogs, indicating that CPN could prevent *H. pylori* infection as a safe and cost-effective anti-adhesive agent.

### 3.3. Effect of CPN on the Colonization of H. pylori in the Mice Gastric Mucosa

In the process of *H. pylori* infection, the successful colonization of *H. pylori* in the gastric mucosa is the first and most important step [18]. Therefore, in order to evaluate the effect of CPN against *H. pylori* infection, the amount of *H. pylori* colonization in the mice gastric mucosa was analyzed. After the adaptive feeding of mice, CPN doses of 200 mg/kg·bw, 400 mg/kg·bw and 600 mg/kg·bw were given by intragastric administration for two weeks. From the third week, in addition to feeding the CPN, 0.4 mL high-concentration *H. pylori* (10^9^ cfu/mL) was fed by intragastric administration every other day for 7 times in total to establish the *H. pylori* infection model. Then, we continued to feed for a week to empty the *H. pylori* that does not colonize in the mice gastric mucosa.

As shown in Table 3, compared with that of the NC group, the number of *H. pylori* increased significantly in the HM group (*p* < 0.05), indicating that the model is successful. Compared with that of the HM group, it was found that CPN intervention significantly reduced the number of *H. pylori* in the mice mucosa (*p* < 0.05). Concerning the number of *H. pylori* in the gastric mucosa of mice in the CPN-200, CPN-400 and CPN-600 groups, they had an *H. pylori* content of 1.63–2.08 × 10^5^ cfu/g. The content of the three CPN groups was significantly lower than that of the HM group (*p* < 0.05), and the CPN-400 group showed the best result (1.63 × 10^5^ cfu/g). It is possible that the CPN showed significant anti-adhesive activity in vitro. A certain dose of CPN could inhibit the colonization of most of the *H. pylori*, so the number of *H. pylori* colonization did not change significantly with increasing CPN concentration. These results indicated that the amount of *H. pylori* colonization in the gastric mucosa could be effectively reduced by CPN intervention. It is possible that the underlying mechanism of the antagonistic effect against *H. pylori* infection is associated with the anti-adhesive effect of CPN.

### 3.4. CPN Alleviates Oxidative Stress with H. pylori Infection-Induced Gastric Injury in Mice

When the mice are infected with *H. pylori*, the *H. pylori* colonizes and multiplies in the stomach. This leads to the release of virulence factors, such as VacA, CagA and urease [19]. These factors then stimulate gastric cells, causing excessive production of oxygen radicals, which in turn increase cellular lipid peroxidation. This process ultimately injures the structure of the cell membrane, leading to injury of the gastric mucosa [20,21]. In order to evaluate the effect of CPN on alleviating oxidative stress induced by *H. pylori* infection, the activities of gastric tissue SOD and GSH-Px and the contents of MDA and LDH were measured. The results are shown in Table 4.

Table 4 shows that compared with the NC group, the activities of SOD and GSH-Px in gastric tissue decreased significantly in the HM group (*p <* 0.05), and MDA as well as LDH contents increased significantly (*p* < 0.05). The activities of SOD and GSH-Px decreased by 40.62% and 35.62%, while MDA and LDH content were elevated by 44.88% and 41.94%, respectively. These results indicated that *H. pylori* infection caused the depletion of antioxidant enzymes (SOD and GSH-Px) and the excessive production of MDA and LDH in the gastric tissue. In other words, *H. pylori* infection led to oxidative stress in the gastric tissue.

SOD is an important endogenous antioxidant enzyme, which could scavenge reactive oxygen species in vivo [22]. Meanwhile, SOD could catalyze the generation of hydrogen peroxide by superoxide anions, reduce the damage of superoxide anions to cells and protect the gastric mucosa from free radical damage. SOD activity of the CPN-400 and CPN-600 groups significantly increased (*p* < 0.05), compared with the HM group. SOD activity of the CPN-600 was group elevated significantly by 53.0% (*p <* 0.05), which was higher than that of positive control group. These results suggest that CPN is effective to alleviate the oxidative stress with *H. pylori* infection-induced gastric injury in mice.

GSH-Px, as an endogenous antioxidant enzyme in the body’s antioxidant system, plays an important role in the antioxidant process [23]. The activity of GSH-Px in the HM group decreased significantly (*p* < 0.05), indicating that *H. pylori* infection could reduce GSH-Px content in gastric tissue. Compared to those of the NC group, GSH-Px activity of the HM group, CPN-200, CPN-400, CPN-600 and positive control groups decreased by 35.62%, 20.22%, 11.61%, 9.95% and 5.46%. Specifically, there was no significant difference between the middle as well as high dose of the CPN groups and positive group. Compared with the HM group, the activity of GSH-Px in the CPN-400 and CPN-600 groups was significantly increased by 37.30% and 39.86% (*p* < 0.05), which was comparable to the positive group, which suggested that CPN intervention significantly increased the GSH-Px level (*p* < 0.05).

MDA is a reactive aldehyde produced after lipid peroxidation of polyunsaturated fatty acids on cell membranes, and it is a marker of oxidative stress. MDA content directly reflects the degree of peroxidation of the biofilm, and excessive accumulation of it will cause the loss of cell membrane function and cause damage to the body [24]. As shown in Table 4, MDA content in the HM group was 289.03 nmol/g, which was 1.45-fold higher than the NC group, indicating that the level of oxidative stress was increased by *H. pylori* infection and led to gastric tissue injury. Compared with the HM group, the lipid peroxidation level of gastric tissue in the CPN-400, CPN-600 and positive control groups was lower, and MDA content decreased significantly by 11.72%, 22.72% and 22.69% (*p* < 0.05). Specifically, the CPN-400 group showed the best effect of all CPN groups, which indicated that CPN could effectively remove free radicals, restore the antioxidant capacity of gastric tissue, and relieve lipid peroxidation on the surface of gastric cell membranes.

LDH is a glycolytic enzyme in the cytoplasm. The action of the LDH that catalyzes pyruvate produces lactic acid in cells. Generally, LDH exists stably in cells. When the gastric tissue is infected by *H. pylori*, the NADPH oxidase in cells is stimulated to generate endogenous stress factors, which attack and lead to lipid peroxidation on the surface of the cell membrane, which destroys the cell membrane, releasing and increasing LDH content. Compared with the NC group, LDH content in the HM group increased significantly by 41.94% (*p* < 0.05), showing that the gastric tissue was injured by *H. pylori* infection, and LDH content was extremely elevated. Compared with the HM group, there was a significant reduction in LDH content following different doses of CPN intervention (*p* < 0.05), of which the CPN-600 group had the best effect, with a reduction of 25.19%, which was close to the level of the NC group and comparable to that of the positive control group. These results indicate that the oxidative stress injury of gastric tissue is significantly alleviated by CPN intervention, and the gastric tissue membrane possesses integrity.

Based on these results, CPN intervention could improve the activities of SOD and GSH-Px, inhibit the degree of lipid peroxidation (MDA), as well as reduce the release of LDH. Therefore, the CPN could improve the total antioxidant capacity of the body and then achieve preventive and protective effects on gastric injury induced by *H. pylori* infection. Some studies have shown that bioactive peptides can alleviate gastric injury by reducing oxidative stress, such as wheat peptides [24] and cod collagen peptides [25]. Similarly, some recent studies indicate that corn peptides (glycopeptides) possess excellent antioxidant activities in vitro, such as metal-ion chelating capacity and free radical scavenging ability [16,26]. Wang et al. reported that glycosylated zein peptides (250 mg/kg·bw) significantly increase endogenous antioxidant enzyme activities and GSH levels and relieve oxidative stress caused by alcoholic liver injury [27]. These results suggested that corn peptides can also reduce oxidative stress, but the protective effect of corn peptides on *H. pylori* infection-induced gastric injury has not been reported.

### 3.5. Effects of CPN on the Expression of Gastric Pro-Inflammatory Cytokines and Chemokines

**Figure 2:**
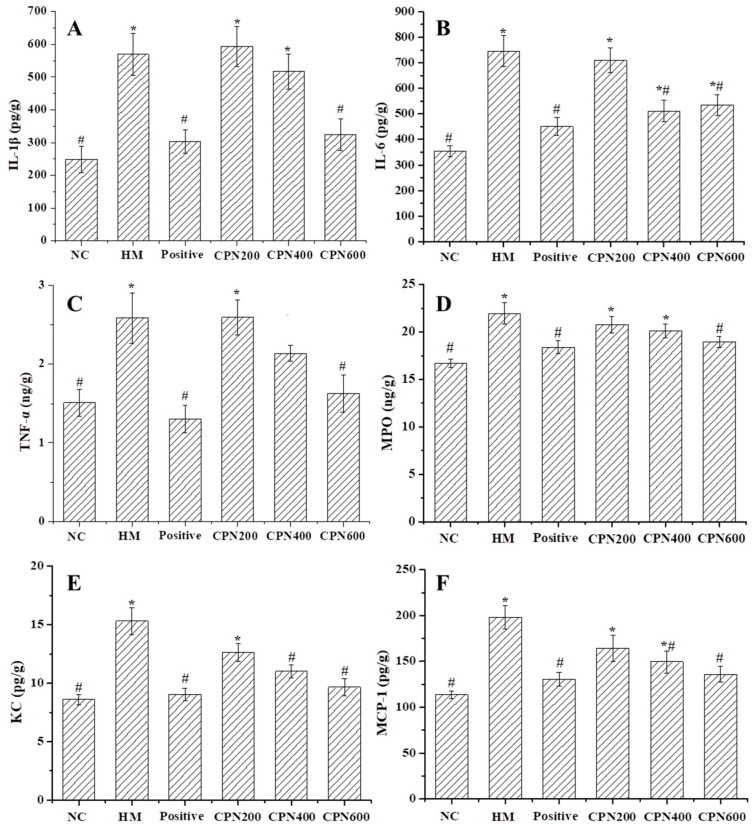
Effects of CPN on gastric contents of IL-1β (**A**), IL-6 (**B**), TNF-α (**C**), MPO (**D**), KC (**E**) and MCP-1 (**F**) in *H. pylori*-infected mice. Data are expressed as the mean ± SD, *n* = 8. * Denotes significant difference to NC group, *p* < 0.05; # denotes significant difference to HM group, *p* < 0.05.

After infecting gastric mucosal epithelial cells, *H. pylori* releases virulence factors that can directly injure the cells, reduce gastric mucus secretion and cause bleeding and other symptoms. The infection also activates the immune system, leading to the production of cellular inflammatory factors such as TNF-α, IL-1β and MPO, which mediate the occurrence of inflammatory reactions [28]. The content of gastric pro-inflammatory cytokines (IL-1β, IL-6, TNF-α and MPO), as well as chemokines (KC and MCP-1), was measured to evaluate the effectiveness of CPN on the inflammatory response induced by *H. pylori* infection. As shown in Figure 2, the contents of IL-1β, IL-6, TNF-α, MPO, KC and MCP-1 in gastric tissue significantly increased in *H. pylori*-infected mice, compared with the NC group (*p* < 0.05), increasing by 128.66%, 110.55%, 72.00%, 31.45%, 77.70% and 74.10%, respectively, which indicated that *H. pylori*-infected mice could abnormally metabolize pro-inflammatory factors and chemokines in the gastric mucosa. These molecules can cause inflammatory gastric injury with mitochondrial dysfunction and increased endoplasmic reticulum stress.

Compared with the HM group, all CPN groups could decrease the contents of IL-1β and IL-6 in the gastric tissue (Figure 2A,B). Specifically, the high-dose CPN could significantly decrease the IL-1β and IL-6 contents by 43.08% and 46.77%, respectively. Additionally, compared with the NC group, there is no significant difference in the effect of high-dose CPN. These results showed that the levels of IL-1β and IL-6 in the gastric tissue were effectively reduced with CPN intervention, and the inflammatory response in the gastric tissue was alleviated. 

As an important cytokine with multiple functions, TNF-α activation is closely associated with a series of pro-inflammatory cytokines, which is related to inflammatory diseases [29]. Studies have shown that the activation of the pro-inflammatory factors (e.g., IL-6 and IL-1β) could increase TNF-α content again, which ultimately aggravates inflammation. TNF-α is one of the strongest pro-inflammatory cytokines; thus, it is often used as an important indicator to judge the degree of inflammation [30]. As shown in Figure 2C, CPN intervention could effectively reduce the TNF-α content in a dose-dependent manner compared with the HM group, while only high-dose CPN could significantly decrease the TNF-α content close to the normal level (*p* < 0.05). 

MPO is a heme protein, and it exists in neutrophils. Neutrophils are stimulated by external factors, which aggregate and release MPO. The activation and infiltration of neutrophils are involved in the gastric inflammation that can be assessed by the increased MPO content [31]. Consequently, MPO is a crucial indicator for assessing the extent of neutrophil infiltration and inflammation. MPO content in the HM group, CPN-200, CPN-400, CPN-600 and positive control groups was 1.31-, 1.24-, 1.20-, 1.13- and 1.10-fold higher compared with that of the NC group, respectively, indicating that *H. pylori* infection caused a significant increase in MPO content in the gastric tissue (*p* < 0.05). This finding is consistent with previous reports [32]. Compared with the HM group, all dose groups could reduce the MPO content, but only the CPN-600 group had a significant difference (*p* < 0.05), decreasing by 13.67%, and compared with the NC group, there was no significant difference. These results showed that treatment with varying doses of CPN effectively improved the infiltration of neutrophils, leading to a reduction in MPO release. This resulted in the alleviation of gastric mucosal injury and inflammation, ultimately maintaining the integrity of the gastric mucosa. A similar result was reported [32]. Li et al. also found that tricholoma matsutake-derived peptides have the ability to counteract alcohol-induced acute gastric mucosal injury by decreasing the MPO levels in gastric tissue [33]. This suggests that CPN intervention can alleviate gastric mucosal injury by reducing the MPO content in the gastric tissue.

The gastric epithelial cytoskeleton rearrangement is induced by *H. pylori* infection, which activates NF-κB, and the expression and release of chemokines (MCP-1, KC) are induced, which lead to gastric epithelial cell apoptosis and mucosal injury [33]. KC and MCP-1 are two important chemokines. Currently, many types of cells, such as macrophages, endothelial cells and smooth muscle cells are known to be affected by chemicals or bacteria, being induced to secrete chemokines [30]. The studies have reported that chemokines have chemotactic activity on cells, which activates monocytes and macrophages, which increase the concentration of Ca^2+^ in the cytoplasm and release oxygen free radicals and lysozyme, up-regulating pro-inflammatory cytokines [34]. The effects of CPN on the content of KC and MCP-1 are illustrated in Figure 2E, F. Compared with the NC group, the KC and MCP-1 content of the HM group increased significantly (*p* < 0.05) from 8.61 ± 0.43 and 113.71 ± 4.26 pg/g to 15.31 ± 1.13 and 197.97 ± 12.43 pg/g, indicating that the chemokine content was greatly elevated by *H. pylori* infection. Compared with the HM group, the KC and MCP-1 contents of three dose groups gradually decreased in a dose-dependent manner, and the CPN-400 and CPN-600 groups showed a significant decrease in KC and MCP-1 contents by 27.97% and 36.93% and 24.46% and 31.33%, respectively. Additionally, the content of the high-dose group was close to the normal level and the CPN effects were comparable to the positive control. The results showed that the possible underlying mechanism of the antagonistic effect against *H. pylori* infection is associated with the anti-inflammatory property of CPN. 

Based on these results, this study found the dose-dependent attenuation of these levels following CPN intervention. In particular, the level of pro-inflammatory cytokines and chemokines returned to normal with the CPN-600 treatment. In other words, CPN intervention could down-regulate the production of chemokines and pro-inflammatory cytokines, which block the development of inflammation and alleviate a series of metabolic changes linked to inflammation, such as oxidative stress and lipid peroxidation, which exert gastric protective effects against *H. pylori* infection. Kan et al. reported a novel wheat peptide that could reduce ethanol-induced gastric mucosal injury through antioxidant and anti-inflammatory activity [24]. These results indicated that the CPN possesses anti-inflammatory properties, which may be an underlying mechanism of the protective effect inhibiting *H. pylori* infection.

### 3.6. Effects of CPN on Key Metabolites in NF-κB Signaling Pathway

**Figure 3:**
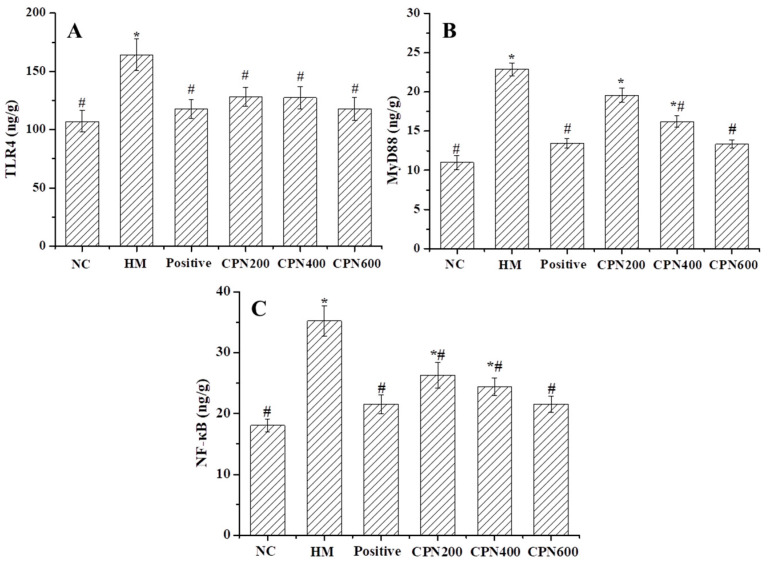
Effects of CPN on gastric contents of TLR4 (**A**), MyD88 (**B**), NF-κB (**C**) in H. pylori-infected mice. Data are expressed as the mean±SD, *n* = 8. * Denotes significant difference to NC group, *p* < 0.05; # denotes significant difference to HM group, *p* < 0.05.

NF-κB plays a key role in regulating the immune response to *H. pylori* infection [35]. The pattern recognition receptor TLR4 of gastric mucosal epithelial cells’ surface can specifically recognize the virulence factors released by *H. pylori*, which promote the increased aggregation of MyD88 and the formation of TLR4/MyD88 complexes, which activate the signaling pathway, leading to an excessive increase in NF-κB content, resulting in an inflammatory reaction [36,37]. Effects of CPN on the important metabolites TLR4, MyD88 and NF-κB in the NF-κB signaling pathway are shown in Figure 3. TLR4, MyD88 and NF-κB contents in the HM group significantly increased (*p* < 0.05) by 53.36%, 108.18% and 95.39%, respectively, compared with the NC group. It showed that *H. pylori* infection promoted the overexpression of TLR4 receptor and MyD88, which activated the NF-κB signaling pathway and caused an inflammatory response. Compared with the HM group, the levels of TLR4, MyD88 and NF-κB in all CPN groups were significantly decreased (*p* < 0.05), indicating that CPN was effective in reducing the levels of TLR4, MyD88 and NF-κB as well as alleviating the inflammatory response. It is worth noting that TLR4 and NF-κB levels were significantly lowered in the 200 mg/kg·bw dose (*p* < 0.05). Moreover, compared with the NC group, the TLR4, MyD88 and NF-κB levels were not significantly different. These results showed that CPN might suppress expression of inflammatory mediators by inhibiting NF-κB activation in the gastric tissues of *H. pylori*-infected mice, which may be another possible mechanism of the protective antagonistic effect of *H. pylori* infection. The previous reports indicated that artemisinin has the potential to reduce the production of ROS induced by *H. pylori* and effectively inhibit the activation of the NF-κB signaling pathway, thus preventing gastric carcinogenesis [34], and Kim et al. found that the astaxanthin could inhibit NF-κB activation of the *H. pylori* in gastric cells by down-regulating TLR4 and MyD88 contents [38]. These results showed that the inhibition of the NF-κB signaling pathway activation is extremely important to prevent *H. pylori* infection-induced gastric injury. Moreover, more studies are required to elucidate the exact mechanism by which CPN inhibits NF-κB signaling pathway activation.

### 3.7. CPN Antagonism H. pylori-Induced Histological Changes in Gastric Tissue

**Figure 4:**
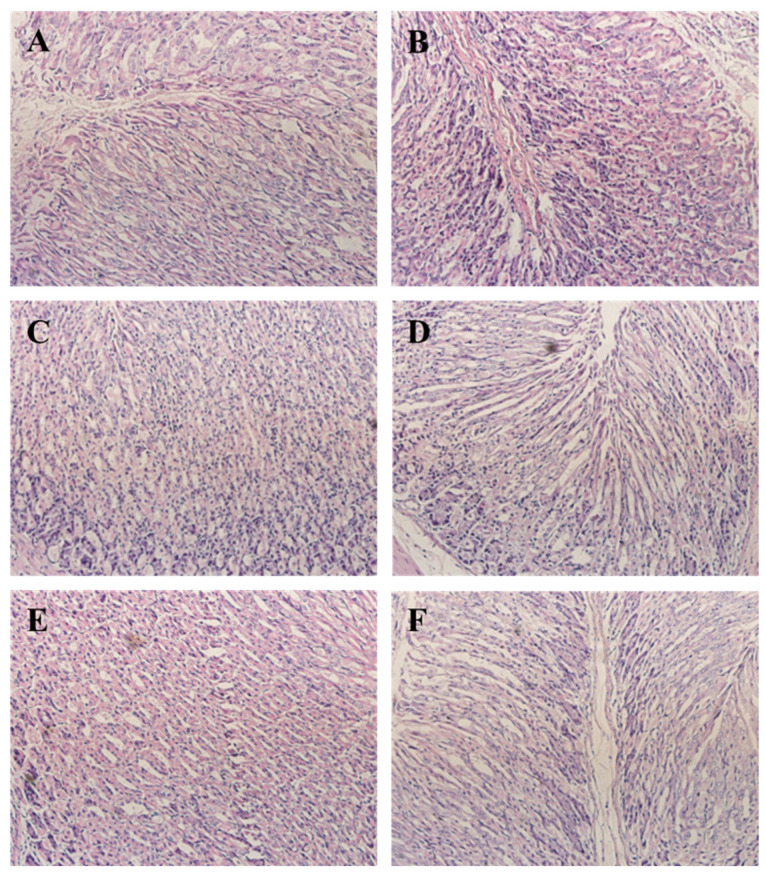
Histopathological sections of the gastric mucosa (H&E, ×400). (**A**) NC group; (**B**) HM group; (**C**) CPN-200; (**D**) CPN-400; (**E**) CPN-600; (**F**) positive control.

*H. pylori* infection induced macroscopic morphological changes in gastric tissue. Compared with the NC group, the HM group had mucosal injury characterized by neutrophil infiltration, whereas the gastric mucosal regions of the NC group showed neither inflammatory lesions nor neutrophil infiltration (Figure 4); the study demonstrated that *H. pylori* infection induced an inflammatory response in gastric epithelial cells, which corroborates with prior findings on this particular model [39]. The study found that CPN intervention significantly improved the injury of gastric mucosal cells in mice infected with *H. pylori*, compared to the model group. There was still evidence of cell damage and swelling, but it was relieved compared to the model group. As the dose of CPN increased, neutrophil infiltration in the gastric mucosa of the mice decreased significantly, resulting in a weakened inflammatory response. The high-dose group had a similar effect as the positive control group. These pathological results indicate that CPN can inhibit histological changes related to neutrophil infiltration in mice, suggesting that CPN intervention can counteract *H. pylori*-induced gastric mucosal inflammation. These findings are consistent with the detection results of gastric tissue biochemical indicators in mice.

## 4. Conclusions

This study indicated that CPN possesses anti-adhesive activities in vitro and protective antagonistic effects against *H. pylori*-induced gastric injury in vivo, as shown by the decreased amount of *H. pylori* colonization, pro-inflammatory cytokines, chemokines and key metabolites of NF-κB signaling pathway levels and increased antioxidant enzyme contents and mitigation of *H. pylori*-induced pathological changes in gastric mucosa, as well as inhibition of the NF-κB signaling pathway activation when compared with the HM group. Specifically, CPN intervention at 600 mg/kg·bw could significantly alleviate gastric injury induced by *H. pylori* infection parameters, restoring levels to those observed in NC group. Overall, this study indicated that the use of corn protein-derived peptides as food products or drugs could establish preventive strategies against gastric *H. pylori* infection.

## Figures and Tables

**Table 1:** Enzymatic conditions and anti-adhesive activity of hydrolysates.

Enzyme	pH	Temperature (°C)	Anti-Adhesive Activity (%)
Trypsin	8.0	37	12.37 ± 0.61 f
Alcalase	8.5	60	40.72 ± 2.61 c
Protamex	7.0	55	39.79 ± 4.71 c
Neutral	7.0	45	50.44 ± 0.27 a
Protease N	7.0	50	44.16 ± 1.99 b
Protease P	7.0	45	47.44 ± 4.50 b
Protease M	6.0	50	39.93 ± 3.76 c
Papain	8.0	50	0
Rebamipide			22.30 ± 0.62 e

Note: different lowercase letters represent significant differences (*p* < 0.05).

**Table 2:** Sequence information of corn protein.

No.	Sequence	Position in Protein	Protein Name	Accession No. on Uniprot
1	LL	8–9, 11–12, 35–36, 155–156	Zein	P04703
2	LLAL	11–14	Zein	P04703
3	APIA	30–33	Zein	P04703
4	LLPP	35–38	Zein	P04703
5	AIAA	61–64	Zein	P04703
6	VALA	106–109	Zein	P04703
7	LAAL	161–164	Zein	P04703
8	LAAA	181–184	Zein	P04703
9	QQ	104–105, 153–154, 157–158, 199–200, 208–209	Glutelin	P04706
10	QQQ	181–183	Glutelin	P04706

**Table 3:** Animal experimental design and the amount of *H. pylori* colonization in mice gastric mucosa.

Group	1–2 Weeks	3–4 Weeks	5 Weeks	cfu/g Gastric Tissue
NC	0.3 mL saline	0.3 mL saline	0.3 mL saline	0
HM	0.3 mL saline	0.3 mL saline + 0.4 mL *H. pylori*	0.3 mL saline	(1.44 ± 0.42) × 10^7^ c
CPN-200	200 mg/kg·bw	200 mg/kg·bw + 0.4 mL *H. pylori*	200 mg/kg·bw	(2.08 ± 0.93) × 10^5^ a
CPN-400	400 mg/kg·bw	400 mg/kg·bw + 0.4 mL *H. pylori*	400 mg/kg·bw	(1.63 ± 0.75) × 10^5^ b
CPN-600	600 mg/kg·bw	600 mg/kg·bw + 0.4 mL *H. pylori*	600 mg/kg·bw	(1.79 ± 0.77) × 10^5^ b
Positive control ^1^	0.3 mL antibiotic	0.3 mL antibiotic + 0.4 mL *H. pylori*	0.3 mL antibiotic	(0.70 ± 0.31) × 10^4^ d

^1^ Mixed antibiotics: 14.25 mg/kg amoxicillin + 7.15 mg/kg Clarithromycin + 14.2 mg/kg Metronidazole. Different lowercase letters represent significant differences (*p* < 0.05).

**Table 4:** Effects of CPN activities of gastric tissue SOD and GSH-Px and MDA and LDH content.

Groups ^1^	SOD (U/g)	GSH-Px (U/g)	MDA (nmol/g)	LDH (ng/g)
NC	2124.35 ± 102.76 #	3257.91 ± 133.91 #	199.49 ± 7.62 #	111.28 ± 4.83 #
HM	1264.23 ± 89.33 *	2097.31 ± 150.49 *	289.03 ± 10.70 *	157.96 ± 6.59 *
CPN-200	1436.88 ± 107.18 *	2599.14 ± 128.78 *	293.98 ± 9.26 *	137.14 ± 7.65 *
CPN-400	1758.01 ± 107.93 #	2879.65 ± 125.19 #	255.13 ± 11.69 *	133.48 ± 7.47
CPN-600	1934.29 ± 110.75 #	2933.47 ± 179.52 #	223.34 ± 10.84 #	118.16 ± 6.96 #
Positive control	1909.61 ± 66.88 #	3079.95 ± 139.21 #	223.46 ± 8.62 #	102.35 ± 3.14 #

^1^ Data are expressed as the mean ± SD, *n* = 8. * Denotes significant difference to NC group, *p* < 0.05; # denotes significant difference to HM group, *p* < 0.05.

## Data Availability

Data are contained within the article.
